# Protective Effects of *Commiphora erythraea* Resin Constituents Against Cellular Oxidative Damage

**DOI:** 10.3390/molecules161210357

**Published:** 2011-12-14

**Authors:** Maria Carla Marcotullio, Federica Messina, Massimo Curini, Antonio Macchiarulo, Marco Cellanetti, Donata Ricci, Laura Giamperi, Anahi Bucchini, Alba Minelli, Anna Lisa Mierla, Ilaria Bellezza

**Affiliations:** 1 Dipartimento di Chimica e Tecnologia del Farmaco, University of Perugia, via del Liceo, 1-06123 Perugia, Italy; Email: fedmessi@tin.it (F.M.); curmax@unipg.it (M.C.); antonio@chimfarm.unipg.it (A.M.); marco@chimfarm.unipg.it (M.C.); 2 Dipartimento di Scienze della Terra, della Vita e dell’Ambiente (DiSTeVA), University of Urbino “Carlo Bo”, via Bramante, 28-061029 Urbino, Italy; Email: donata.ricci@uniurb.it (D.R.); laura.giamperi@uniurb.it (L.G.); elena.bucchinianahi@uniurb.it (A.B.); 3 Dipartimento di Medicina Sperimentale e Scienze Biochimiche, University of Perugia, via del Giochetto-06124 Perugia, Italy; Email: aminelli@unipg.it (A.M.); annalisam79@gmail.com (A.L.M.); ilaria.bellezza@unipg.it (I.B.)

**Keywords:** *Commiphora erythraea *(Burseraceae), furanodienone, lipid peroxidation, 5-LOX, antioxidant

## Abstract

By bioguided fractionation of the hexane extract of *Commiphora erythraea* resin we isolated four furanosesquiterpenoids that were tested for their protective activity against oxidative stress. Furanodienone and 1,10(15)-furanogermacra-dien-6-ones showed to be potent inhibitors of lipid peroxidation (IC_50_ of ~0.087 μM), being more active than the methoxylated analogues. Furthermore, using BV2 microglial cells, we found that furanodienone from *C. erythraea* is able to counteract LPS-induced cell death and decrease LPS-induced NO generation thus protecting microglial cells from LPS-induced cytotoxicity. Finally, docking studies were undertaken to gain insight into the possible binding mode of the isolated compounds at 5-LOX binding site.

## 1. Introduction

Lipid peroxidation (LPO) can be defined as the oxidative degradation of unsaturated lipids by the action of various reactive oxygen (ROS) and nitrogen (RNS) species that can determine the formation of different products that have been shown to be extremely toxic. LPO proceeds by three distinct mechanisms: Free-radical oxidation, non-radical non-enzymatic oxidation and enzymatic oxidation [1].

Free radical oxidation is initiated by free radical species such as peroxyl, alkoxyl and hydroxyl radicals. Nitric oxide (NO), produced by nitric oxide synthase (NOS), is not directly involved into LPO, but it reacts with superoxide to give peroxynitrite (RNS) which may initiate LPO. The non-radical non-enzymatic oxidation is mostly due to the action of singlet oxygen, while the enzymatic oxidation is due to the action of enzymes such as LOX and COX.

Lipoxygenases, which are non heme iron-containing dioxygenases, oxidize arachidonic acid and linoleic acid to arachidonate hydroperoxide and linoleate hydroperoxide, respectively. Mammalian cells have several LOXs (e.g., 5-, 12-, 15-, and 12/15-LOX), in which the numbers refer to the position where they insert oxygen into arachidonate [2].

5-Lipoxygenase (5-LOX) is one of the key enzymes in the synthesis of leukotrienes, inflammatory eicosanoids [3] that are capable of promoting neurodegeneration [4,5], and other diseases [6,7,8,9].

Neuroinflammation, which is characterised by activation of microglia and subsequent production of proinflammatory cytokines, plays an important role in the neurodegenerative process [10]. Recently many efforts have been focused on the isolation of natural inhibitors of different human lipoxygenases [11,12,13] that can be useful anti-inflammatory agents.

**Figure 1:**
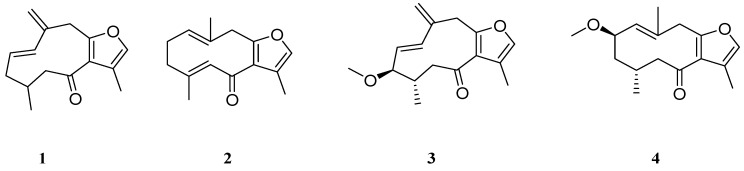
Isolated compounds from *C. erythraea *resin.

Traditionally, *Commiphora* oleo-gum resins are largely used for their biological activity to treat colds, fever, malaria, as antiseptic in skin infections and in wound healing [14]. At the request of the IPO association [15] we undertook the study of the anti-inflammatory activity of *C. erythraea *resin, commonly sold in marketplaces in Ethiopia. Agarsu (*Commiphora erythraea* (Ehrenb.) Engl. resin, Burseraceae) is used in Ethiopia as an anti-inflammatory, antiseptic, against skin infections and it is utilised on livestock against ticks [16]. Phytochemical studies on the composition of agarsu showed that furanosesquiterpenoids **1**–**4** (Figure 1) are the most characteristic components, accounting for more than 60% of the extracts of *C. erythraea *resin [17].

Furanosesquiterpenoids from *C. myrrha* have proved to be good radical scavengers against singlet oxygen [18] and hence can be considered good anti-inflammatory agents. Fraternale and Coll. [17] showed that hexane extract subfraction (H3) containing more of polar furanogermacrenones **3** and **4** acts as antioxidant in a DPPH test, so compounds **3** and **4** can be regarded as good antioxidant compounds. In this study, we report the bioguided isolation of furanodienone **2**, as the most active compound of the *C. erythraea *resin hexane extract in the inhibition of 5-LOX mediated LPO and in reducing LPS-mediated NO production in BV-2 microglial cells.

## 2. Results and Discussion

### 2.1. Lipid Peroxidation Inhibition

To evaluate the activity of *C. erythraea* oils and extracts [17] as inhibitors of 5-LOX, we chose an easy spectrophotometric assay that relates the absorbance with the amount of 5-LOX-induced peroxides, comparing their activity to that of different compounds such as radical scavengers (e.g., BHT, butylated hydroxytoluene), antioxidant (e.g., ascorbic acid) and inhibitors of 5-LOX (e.g., caffeic acid [19]). The screening led once again to the identification of the hexane extract as the most active (Table 1).

**Table 1:** Inhibitory activity of oils, extracts and fractions in 5-LOX assay.

Sample	Lipid peroxidation inhibition ^a^ IC_50_ (μg/mL)	Lipid peroxidation inhibition ^a^ IC_50_ (μM)
HD ^b^	15.120 ± 0.520	=
D ^c^	14.258 ± 1.560	=
SF1 ^d^	17.893 ± 1.090	=
SF2 ^e^	23.757 ± 4.860	=
H ^f^	0.743 ± 0.040	=
H1	56.848 ± 8.360	=
H2	1.365 ± 0.200	=
H3	0.756 ± 0.060	=
**1**		0.091 ± 0.003
**2**		0.087 ± 0.001
**3**		3.381 ± 0.079
**4**		3.263 ± 0.057
BHT	3.860 ± 0.850	17.517 ± 0.850
Caffeic acid	5.764 ± 0.480	31.993 ± 0.480
Ascorbic acid	18.630 ± 1.310	105.780 ±1.310

^a^ The values are the average of three determinations (±s.d.); ^b^ HD: Hydrodistilled oil; ^c^ D: Steam distilled oil; ^d^ SF1: obtained by extraction at 20 MPa and 20 °C for 1 h; ^e^ SF2: obtained by extraction at 100 MPa and 40 °C; ^f^ H: Hexane extract.

The activity of this extract was particularly interesting as it was 5-fold higher than BHT, 25-fold higher than ascorbic acid and 7-fold higher than caffeic acid that are to be selective inhibitors of 5-LOX, so we decided to further investigate it.

The hexane extract was divided by column chromatography into three different fractions (H1, H2 and H3) [17]. The sesquiterpene-containing fraction (H1) did not exhibit 5-LOX inhibition in this assay. Subsequent purification (Scheme 1, Supporting Information) of the active fractions (H2 and H3) led to the isolation of four sesquiterpenoidic compounds as the most abundant, namely 1,10(15)-furanogermacra-dien-6-one (**1**) [20] and furanodienone (**2**) [20,21] from fraction H2 and rel-3*R*-methoxy-4*S*-furanogermacra-1*E*,10(15)-dien-6-one (**3**) [21,22] and rel-2*R*-methoxy-4R-furano-germacra-1(10)*E*-en-6-one (**4**) [21,22] from H3, that resulted pure by GC-MS, TLC and NMR analysis (Figures S1, S2 and S3, Supplementary Information). 5-LOX inhibition activities of the isolated compounds are shown in Table 1. It is evident the remarkable inhibitory activity of these compounds, particularly of compounds **1** and **2**, that are forty times more active than the methoxylated ones.

### 2.2. Inhibition of RNS formation

**Figure 2:**
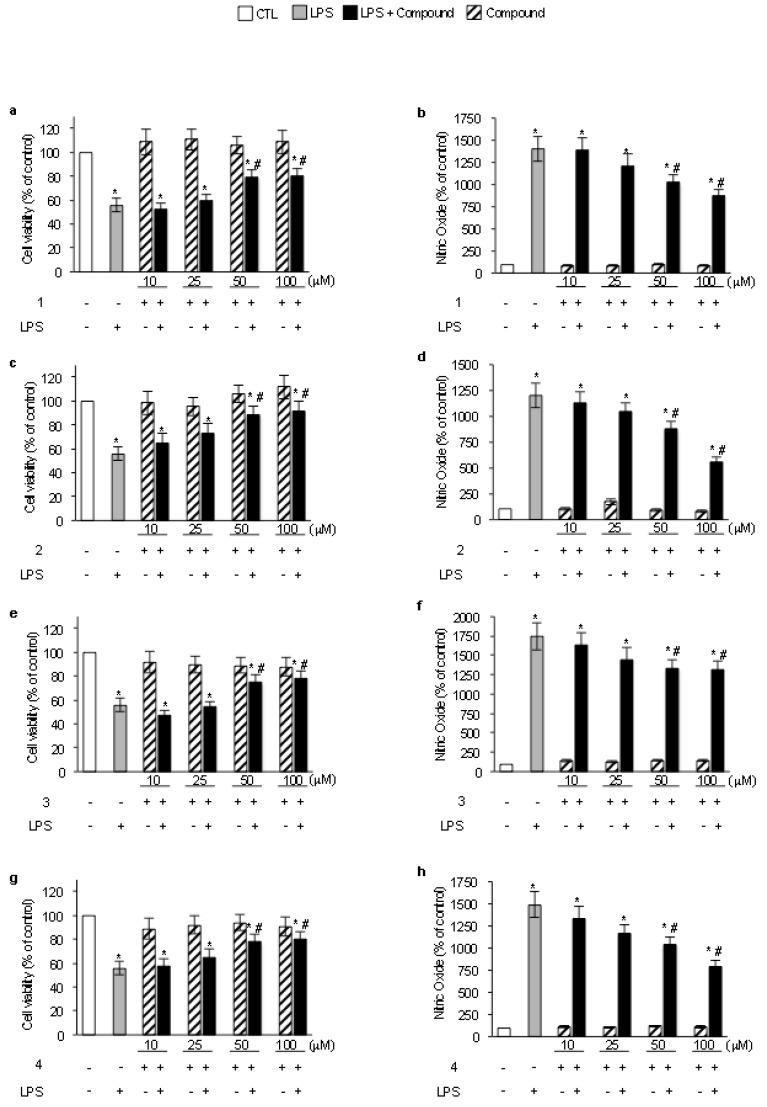
The compounds reduced LPS-induced NO generation and protected microglial cells from LPS-induced cytotoxicity. BV-2 microglial cells were treated for 24 h with the tested compounds (50μM), then stimulated with 10μg/mL LPS for further 24 h and used to assess cell viability (a, c, e and g) and NO levels (b, d, f and h). Control values (mean ± S.D., n = 4) are given as 100% * p < 0.05 *vs.* control cells; ^#^ p < 0.05 *vs.* LPS-treated cells.

To determine the activity against RNS production, we investigated the effects of isolated compounds on cell viability and NO production in LPS-exposed BV-2 microglial cells. LPS from Gram-negative bacteria is a prototypical trigger of inflammation [2], commonly used to test the activity of anti-inflammatory compounds. A 24 h exposure to 10 μg/mL LPS reduced the percentage of viable cells to 56.3 ± 6%. A 24 h pre-treatment with each compound restored cell viability in a concentration dependent manner with different EC_50_ values, *i.e.*, compound **1**: 48.68 μM; compound **2**: 32.20 μM; compound **3**: 55.43 μM; compound **4**: 50.4 μM (Figure 2a, c, e and g).

Under the same experimental conditions, LPS induced a 10-fold increase in NO generation that was reduced by all the tested compounds in a concentration dependent manner (Figure 2b, d, f, and h). The compounds, by themselves, did not affect either cell viability or NO generation. These data show that all the compounds decrease LPS-induced NO generation thus protecting microglial cells from LPS-induced cytotoxicity, and that compound **2** was the most effective in reducing NO levels and restoring cell viability. Data clearly show that all the isolated compounds, especially furanodienone **2**, can reduce the formation of lipid peroxides and counteract LPS-induced cytotoxicity and nitiric oxide generation.

### 2.3. Molecular Docking

We have previously shown [17] that fraction H2 did not provide a remarkable scavenging effect as determined by the DPPH test, while fraction H3 was the most effective (EC_50_= 12.872 ± 1.75 mg/mL *vs*. 3.487 ± 0.30 mg/mL, respectively; Trolox: EC_50_ = 0.011 ± 0.001 mg/mL). These results led us to hypothesize that our observations are based on a non-redox mechanism. Thus, we developed a homology model of 5-LOX that was instrumental to investigate the binding mode of our compounds to the enzyme binding site.

**Figure 3:**
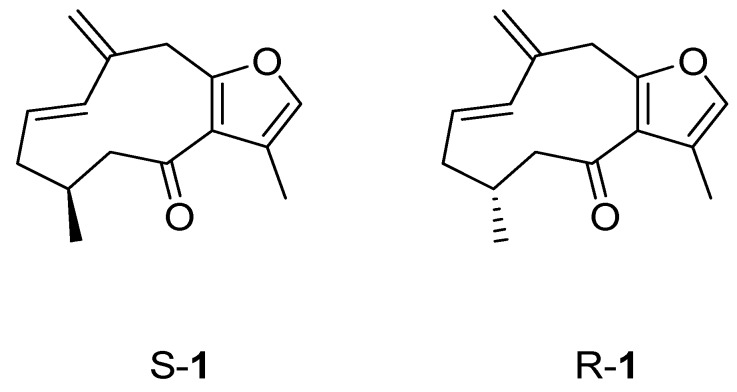
Structures of the two diasteroisomers of compound **1**.

Like other enzymes belonging to the lipoxygenases superfamily [24], the model of human 5-LOX consists of two structural domains (Figure 3): A small N-terminal β barrel domain, and a larger catalytic domain containing an iron atom bound to His368, His373, His551, Asn555 and the terminal carboxylic group of Ile674. The side chain of Asn555, in particular, constitutes the fifth coordination position of the iron, whilst the sixth coordination position faces an open cavity wherein the substrate binds the enzyme. This latter cavity was used as a binding site to dock our series of compounds using an induced fit docking (IFD) procedure as reported in the method section. During docking experiments, both the *S* and *R* isomer of compound **1** (*S*-**1** and *R*-**1**; Figure 3) were docked into the binding site.

**Figure 4:**
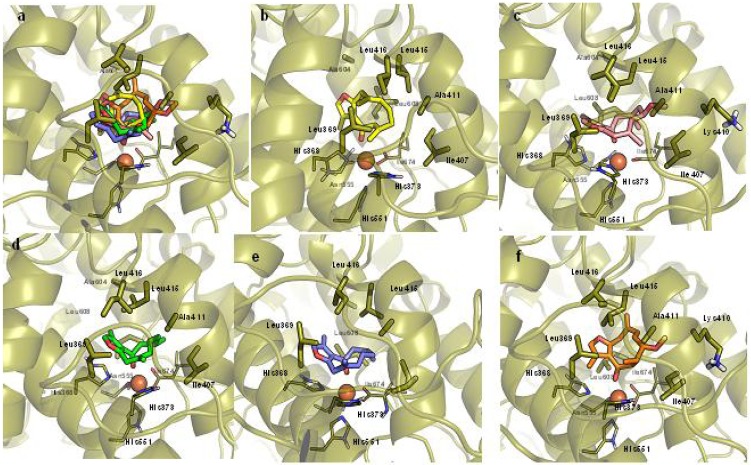
Selected binding poses of compounds **1**, **2**, **3** and **4** into the catalytic cleft of 5-LOX. Overlay of the compounds (a); compound **2** (b); compound **4** (c); compound *S*-**1** (d); compound *R*-**1** (e); compound **3** (f).

Briefly, IFD takes into account conformational rearrangements of the side chains of residues, lining the binding site that may occur upon ligand binding. Among the possible docking solutions, the binding mode of compounds **1**, **2**, **3** and **4** to human 5-LOX was selected as the docked pose presenting the best IFD score and the closest proximity to the iron atom. Accordingly, the selected binding poses are reported in Figure 4, whilst Table 2 reports the relative docking scores as crude estimation of the binding energies.

**Table 2:** Selected docking solutions of compounds along with energetic scores (Glide and IFD scores).

Code	IC_50_ (μM)	Glide Score (kcal/mol)	IFD Score
*S*-**1**	0.091 ± 0.003 ^a^	−8.01	−1067.53
*R*-**1**	0.091 ± 0.003 ^a^	−7.73	−756.54
**2**	0.087 ± 0.001	−9.39	−757.63
**3**	3.381 ± 0.079	−9.55	−1094.74
**4**	3.263 ± 0.057	−8.59	−789.2

^a^ The IC_50_ value is referred to the undetermined natural stereoisomer.

Figure 4 shows that all compounds adopt a conserved pattern of interactions with 5-LOX. This includes a coordination bond of the carbonyl group to the iron atom as well as multiple hydrophobic interactions with the side chains of Leu369, Ile407, Ala411, Leu415, Ile416, Ala604 and Leu608. For compounds *S*-**1**, *R*-**1**, **3** and **4**, an aromatic π-π interaction can be also observed between the furan moiety and the side chain of His368. Remarkably, residues interacting with our compounds are in agreement with those depicted by early works as pivotal to ligand binding in the catalytic cleft of 5-LOX [25,26].

The analysis of Table 2, however, reveals the lack of a clear trend between the inhibition activity of the compounds and the docking scores, suggesting the weakness of the IFD scoring function to discriminate between active and poor binders of 5-LOX. On the one hand, it is likely that this may be due to a poor parameterization of the scoring function that is unable to correctly assess the binding energy of the coordinative bond of the carbonyl group to the iron atom.

On the other hand, an additional issue could be the underestimation of the enthalpic and entropic effects related to the conformational profiles of our compounds. In this context, we deemed it of interest to address part of these issues by carrying out thorough conformational studies on the compounds. The aim of this part of the work was to identify the global minimum conformation of each compound and compare its energetic state to that of the relative bioactive conformation. In order to explore the largest conformational space of the cyclic compounds, we performed stochastic dynamic simulations at high temperature. Then, a conformational sampling was carried out, selecting different conformers at regular intervals for further analysis. Finally, the selected conformers were submitted to further conformational refinements using the Monte Carlo Multiple Minimum (MCMM) method.

Table 3 reports the conformational energy values of the bioactive conformations and the global minimum conformation as found for each compound, whilst Figure 4 shows a superposition between the two conformations that evidences the differences in the relative shapes.

In agreement with the inhibition activities, the analysis of Table 3 shows that the conformational gap energy between the global and bioactive conformation is relatively high for the least active compounds (**3** and **4**). This observation may be ascribed to conformational stretching induced by steric hindrances between the methoxylic group of such compounds and the side chain of Ala411 (Figure 4), that force the molecule towards a high energy and less populated bioactive conformation.

Conversely, the most active compounds (**1** and **2**) show low conformational gap energies. Remarkably, the *S* isomer of compound **1** is endowed with a more stable bioactive conformation than the *R* isomer, suggesting that it is the eutomer for the inhibition of 5-LOX.

**Table 3:** Conformational energy values of tested compounds.

Code	Global Minimum(Kcal/mol)	Bioactive Conformation (Kcal/mol)	Conformational Gap Energy (Kcal/mol)
*S*-**1**	45.88	46.53	+0.65
*R*-**1**	45.88	49.69	+3.81
**2**	66.98	66.99	+0.01
**3**	41.99	53.53	+11.54
**4**	47.34	50.45	+3.11

## 3. Experimental

### 3.1. General Procedures

NMR spectra were recorded using a Bruker Avance DPX-400 spectrometer operating at frequencies of 400 MHz (^1^H) and 100.62 MHz (^13^C). The spectra were measured in CDCl_3_. The ^1^H- and ^13^C-NMR chemical shifts (δ in ppm) were referenced to the signal of the solvent 7.26 (^1^H) and 77.00 (^13^C). H-H COSY, NOESY, HMQC, and HMBC were used to assign the structures. IR spectra were recorded on a JASCO V-530 spectrophotometer. Column chromatography was performed using Davisil LC60A 60–200 μm silica gel. Preparative TLC were performed using Merck Silica gel 60 F_254_ 0.5 mm plates. Fractions were monitored by TLC (Silica gel 60 F_254_, Merck) and spots on TLC were visualized under UV light and after staining with p-anisaldehyde-H_2_SO_4_-EtOH (1:1:98) followed by heating at 110 °C. Soybean 5-Lipoxygenase (EC 1.13.11.12, Type V), RPMI, FCS, penicillin, streptomycin, LPS and MTT were purchased from Sigma.

### 3.2. Material

The resin of *Commiphora erythraea* (Agarsu grade I) commercialized by Agarsu Liben Cooperative and imported into Italy by the IPO (Increasing People Opportunities) association was studied. A voucher specimen (# MCM-1) of the resin (Agarsu grade I) is deposited at the Dipartimento di Chimica e Tecnologia del Farmaco - Sez. Chimica Organica, University of Perugia.

### 3.3. Extraction, Isolation and Identification of Tested Compounds

Preparation of oils and extracts has been reported [17] and fractionation procedures [17] of the hexane extract have been already reported and are summarized in Scheme S1 (Supplementary Material).

### 3.4. Determination of Lipid Peroxidation Inhibitory Effect

Inhibition of the enzyme activity was assayed spectrophotometrically according to Holman. This method was modified by Sud’ina *et al.* [28]. Briefly, the assay mixture (1 mL final volume) contained 50 mM sodium phosphate, 10 mM linoleic acid (10 μL), and the sample (or the same quantity of solvent as reference), pH 6.8. The mixture was maintained at 23 °C for 10 min, then 0.18 μg mL^−1^ commercial soybean 5-LOX was added and the formation of hydroperoxides was recorded spectrophotometrically at 235 nm. The IC_50_ values, defined as the amount of antioxidant necessary to inhibit lipid peroxidation by 50%, were calculated from the results.

### 3.5. Cell Cultures and Viability

Murine microglial BV-2 cells were cultured in RPMI (Life Technologies (GibcoBRL, Gaithersburg, MD, USA) supplemented with 10% Foetal Bovine Serum (FBS), penicillin (100 U/mL), and streptomycin (100 µg/mL) at 37 °C in a humidified incubator under 5% CO_2_. After 24 h subculture, cells (9 × 10^4^) were incubated for 24 h with the compounds dissolved in DMSO (stock solutions, 500 mM), and then exposed to 10 μg/mL lipopolysaccharide (LPS, *Escherichia coli* serotype 0111:B4, Sigma-Aldrich, St. Louis, MO, USA) for the indicated times. Cell viability was assessed by the conventional MTT (3-[4,5-dimethylthiazol-2-yl]-2,5-diphenyltetrazolium bromide) reduction assay after washing the cells with PBS (phosphate-buffered saline). Results were expressed as the percentages of reduced MTT, assuming the absorbance of control cells as 100%. EC_50_ values were determined graphically.

### 3.6. Measurement of NO Production

Nitric oxide (NO) production was determined indirectly through the measurement of nitrite, a stable metabolite of nitric oxide, by the Griess reaction. The absorbance was read at 550 nm using a microtiter plate reader (SAEC, Florence, Italy) and results, expressed as % of the control, were normalised to cell viability. Nitrite standard reference curve was prepared for each determination.

### 3.7. Statistical Analysis

All results were confirmed in at least three separate experiments and expressed as mean ± S.D. Data were analyzed for statistical significance by Student’s *t*-test. *p*-values < 0.05 were considered significant.

### 3.8. Molecular Modelling

Homology modelling techniques were applied to predict the structure of human 5-LOX. This was accomplished using the Swiss-Model server [29]. The procedure consisted in running a sequence similarity search with the human 5-LOX (Uniprot database: sequence id P09917) [30] against a database of known protein structures (PDB database) [31] with default parameters. After the identification of the closest homolog sequence in the structure of 8R-LOX from *Plexaura homomalla* (40% sequence similarity; pdb code: 2FNQ) [32], a pairwise sequence alignment was generated between the target sequence (5-LOX) and the template sequence (8R-LOX). On the basis of such alignment, a 3D-model of 5-LOX was finally generated.

The homology model of 5-LOX was then submitted to geometrical and energetic refinements using the Protein Preparation protocol as implemented in the Schrödinger package [33]. Briefly, it consisted in an early optimization of the hydrogen bond network within the protein, and a minimization of the whole protein structure with the OPLS-2001 force field [34]. During the minimization stage, in particular, restrains on the atomic coordinates of the protein were set to allow a maximum of 0.3Å root mean square deviation (RMSD) from the original values. The final refined structure of 5-LOX was endowed with a RMSD of 3.66 Å from the template structure (2FNQ). This value was calculated using the coordinates of the backbone atoms between the catalytic sites of the two enzymes. The quality of the model was assessed by different statistical and geometrical validation methods using Molprobity and Verify3D servers (Figure S4, Supplementary Material) [35,36].

Before running docking experiments, all ligands were prepared with the ligand preparation tool implemented in the Schrödinger Suite 2010. In particular, we considered for each compound different tautomers and ionization states at the physiological pH 7.0. The binding site for docking experiments was identified using Sitemap (SiteMap, version 2.4-Schrödinger Suite) as the larger cavity around the iron atom. The Induced Fit Docking (IFD) protocol implemented in the Schrödinger suite (Induced Fit Docking protocol-Schrödinger Suite) was used for docking experiments with the aim of taking into account ligand-induced conformational adaptations of the binding site. The IFD protocol develops in three steps. In the first one, a Glide (Glide version 5.6-Schrödinger Suite) docking procedure is performed using a softened potential (van der Waals radii scaling) and temporarily removing side chains within 3Å from the docked ligand by mutating them into alanines. The second step is represented by the reconstruction of the removed side chains by predicting their most energetic favoured conformation and the minimization of the edited residues using Prime (Prime version 2.2-Schrödinger Suite). In the third and final step, each ligand is docked again into the resulting induced-fit enzyme using the Glide Extra Precision (XP) protocol. During docking experiments, the formal charge of the iron atom was set to +3. Exhaustive conformational analyses of the compounds were performed using stochastic dynamic simulations at high temperature and the Monte Carlo Multiple Minimum (MCMM) method. These studies were performed using Macromodel software (MacroModel version 9.8-Schrödinger Suite) and the MMFF94s force field. The GB model was used for the implicit treatment of solvent [37]. During stochastic dynamic simulations, each compound was submitted to an incremental temperature as it follows: (i) 10 ps at 300 °K for the equilibration step; (ii) 100 ps at 300 °K followed by 100 ps at 600 °K for the heating step; (iii) 1,000 ps at 900 °K for the production step. Ten conformers for each compound were sampled at regular intervals and submitted to the Monte Carlo Multiple Minimum (MCMM) [38] method for further conformational refinements. The MCMM method consists of multiple cycles of three steps each: (i) a stochastic generation of a new conformation; (ii) the energetic minimization using the truncated Newton conjugate gradient method [39]; (iii) the comparison with minimum conformers found in previous cycles. Concerning the latter step, the new minimum conformation is discarded if the RMSD of the atomic coordinates is lower than 0.5Å from other previously generated minimum conformations.

## 4. Conclusions

It is known that LOX are sensitive to antioxidant due to a limited availability of lipid hydroperoxides necessary for the catalytic cycle of the enzyme. Although like in a DPPH test (EC_50_ = 12.872 ± 1.75 mg/mL *vs* 3.487 ± 0.30 mg/mL) [17], fraction H2 showed a lower LPO inhibitory activity than H3, the difference was not so remarkable. These results suggest that a scavenging effect may not be a critical factor behind 5-LOX inhibition and the antioxidant effect could be due to a specific interaction of the constituents with the enzyme. Bioguided separation of the hexane extract of *C. erythraea* led to the isolation of four sesquiterpenoids that had been previously found in different *Commiphora* spp. and that showed, *in vitro*, a protective effect against oxidative damage by reducing lipid peroxidation and NO production. Two of them, namely furanogermacradienones **1** and **2**, displayed an interesting antioxidant profile and their higher activity respect to **3** and **4** could be explained with a steric hindrance of the methoxy group at the catalytic site of LOX. Furthermore, in this study we demonstrated that compound **2** is particularly able to counteract LPS-induced cytotoxicity and nitiric oxide generation. Compound **2** already showed COX-2 and COX-1 inhibitory activity [27] and these investigations put in evidence that it should be deeply studied further to develop a new anti-inflammatory compound.

## Supplementary Materials

Supplementary material can be accessed at: http://www.mdpi.com/1420-3049/16/12/10357/s1.
