# Supplementary material for: Protective Effects of Commiphora erythraea Resin Constituents Against Cellular Oxidative Damage
Source: Molecules. 2011 Dec 14;16(12):10357–69. doi: 10.3390/molecules161210357 (PMC6264320; doi:10.3390/molecules161210357)
Supplement: Supplementary file 1 [file molecules-16-10357-s001.doc]

**Protective Effects of *Commiphora erythraea* Resin Constituents on Cellular Oxidative Damag*e*.**

Maria Carla Marcotullio^a^,*, Federica Messina^a^, Massimo Curini^a^, Antonio Macchiarulo^b^, Marco Cellanetti^b^, Donata Ricci^c^, Laura Giamperi^c^, Anahi Bucchini^c^, Alba Minelli^d^, Anna Lisa Mierla^d^, Ilaria Bellezza^d^.

*^a^Dipartimento di Chimica e Tecnologia del Farmaco-sez. Chimica Organica, University of Perugia, via del Liceo, 1-06123 Perugia, Italy.*

*^b^Dipartimento di Chimica e Tecnologia del Farmaco-sez. Chimica Farmaceutica 1, University of Perugia, via del Liceo, 1-06123 Perugia, Italy.*

*^c^Dipartimento di Scienze della Terra, della Vita e dell’Ambiente (DiSTeVA)-sez. Biologia Vegetale, University of Urbino “Carlo Bo”, via Bramante, 28-061029 Urbino, Italy.*

*^d^Dipartimento di Medicina Sperimentale e Scienze Biochimiche-sez. Biochimica Cellulare, University of Perugia, via del Giochetto-06124 Perugia, Italy.*

**Corresponding author**: Maria Carla Marcotullio-Dipartimento di Chimica e Tecnologia del Farmaco-sez. Chimica Organica-Via del Liceo, 1-06123 Perugia-Italy

Tel.: +39-075-5855107; Fax: +39-075-5855116; email: [marcotu@unipg.it](mailto:marcotu@unipg.it)

**Scheme S1:** Scheme of the chromatographic purification of the hexane extract. Column chromatography was performed using Davisil LC60A 60-200 μm silica gel. Preparative TLC were performed using Silica gel 60 F_254_, 0.5 mm plates.

PE (Petroleum ether), DCM (Methylene chloride), EA (Ethyl acetate), EE (Ethyl ether)

**Preparative TLC of H2-3 (50 mg) Preparative TLC of H2-5 (25 mg)**

Eluent: PE-DCM-Acetone (50:50:1) Eluent: PE-EA (7:3)

H2-31: 10 mg (**1**) H2-51: 5 mg (**2**)

H2-32: 5 mg H2-52: 2 mg (**5**)

H2-33: 3 mg H2-53: 10 mg (**3**)

H2-34: 15 mg (**2**)

**Figure S1**: Mass spectra of tested compounds: **a)** Compound **1**, **b)** Compound **2**; **c)**  Compound **3**; **d)**  Compound **4**.

**Instrument and condition of the elution**: Analyses were performed on a Hewlett Packard HP 6890 gas chromatograph. The GC instrument was equipped with a Hewlett Packard MS 5973 mass selective detector and a fused silica capillary column (HP-5MS; 30 m x 0.25 mm i.d., 0.25 μm film thickness). The oven temperature was programmed from 70 °C for 4.50 min, then ramped at 10 °C/min to 270 °C, and held for 20 min. Injector and detector temperatures were 250 and 270 °C, respectively. Samples were dissolved in methylene chloride to give 1% w/v solutions and were injected in the splitless mode using helium as carrier gas (1 mL/min); the injection volume was 1 μL. The ionization energy was 70 eV.

**a)
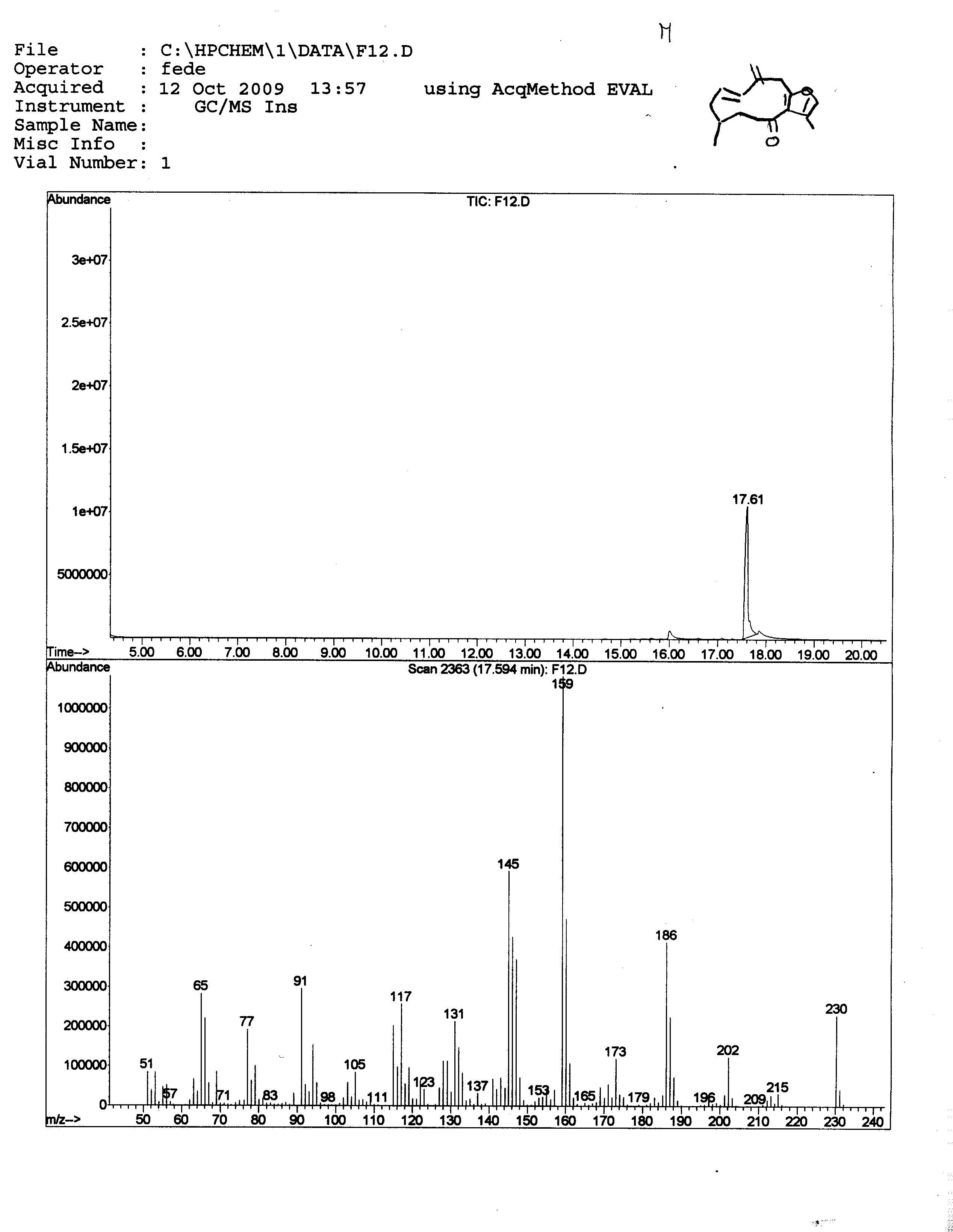
**

**b)
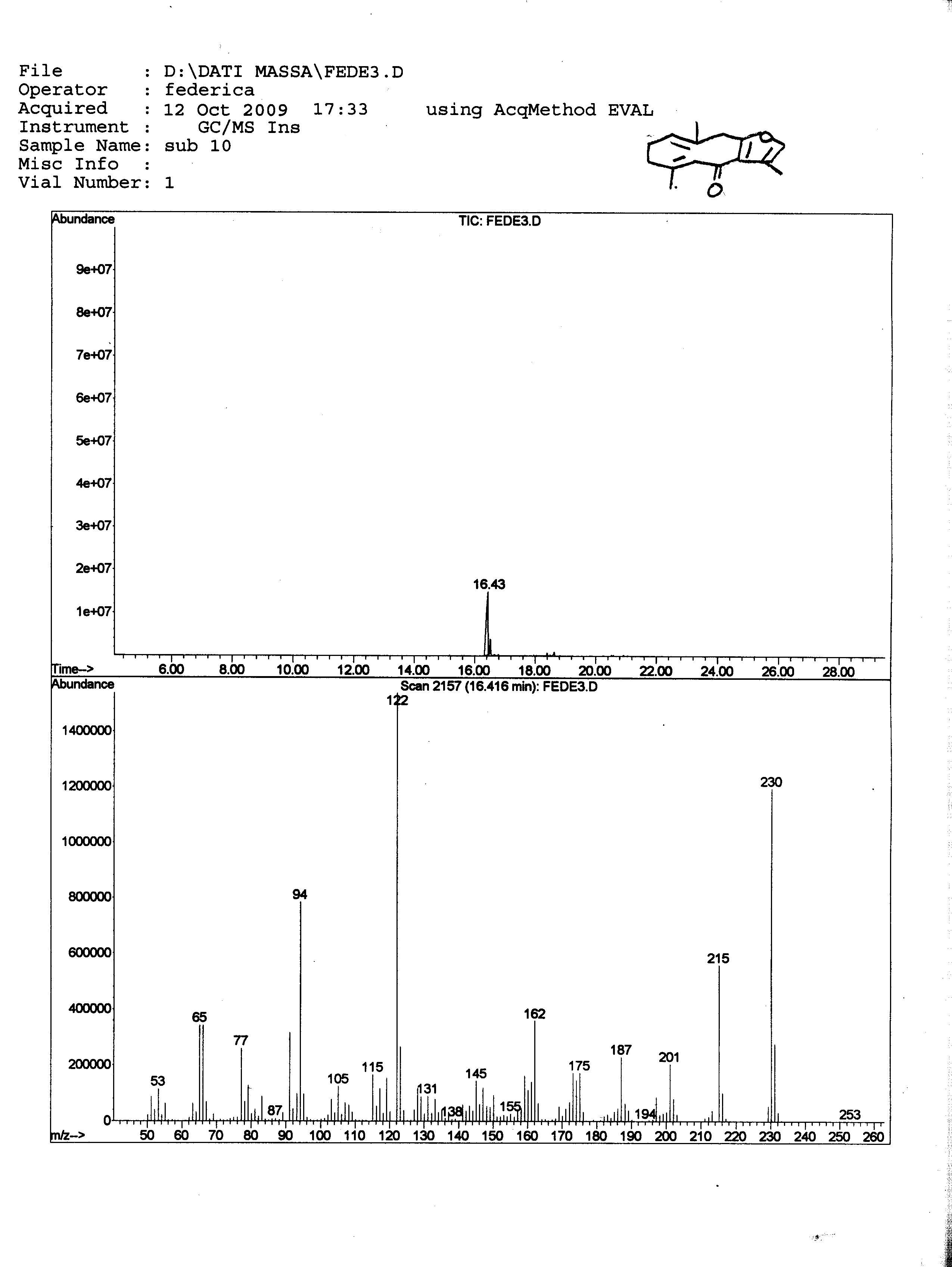
**

**c)
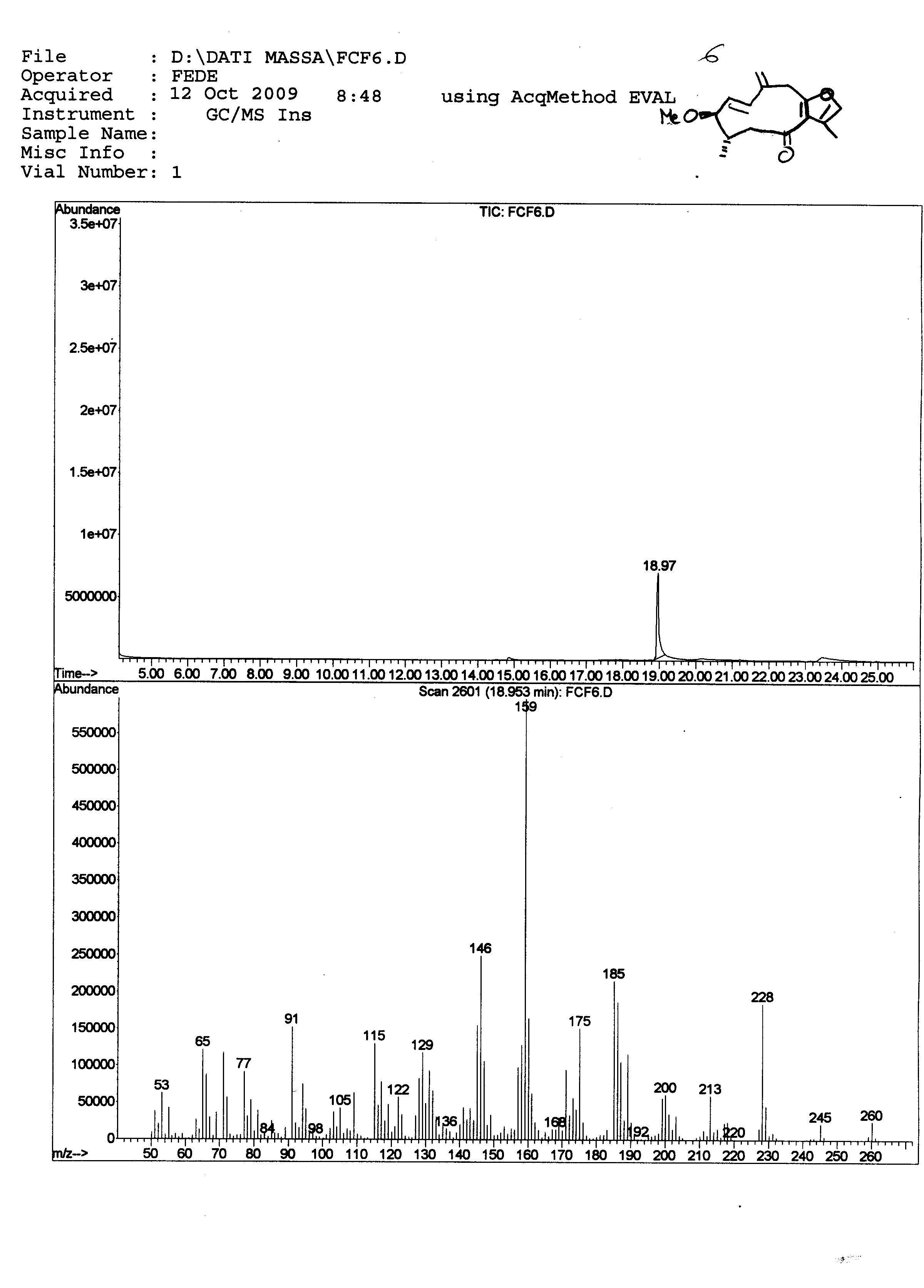
**

**d)
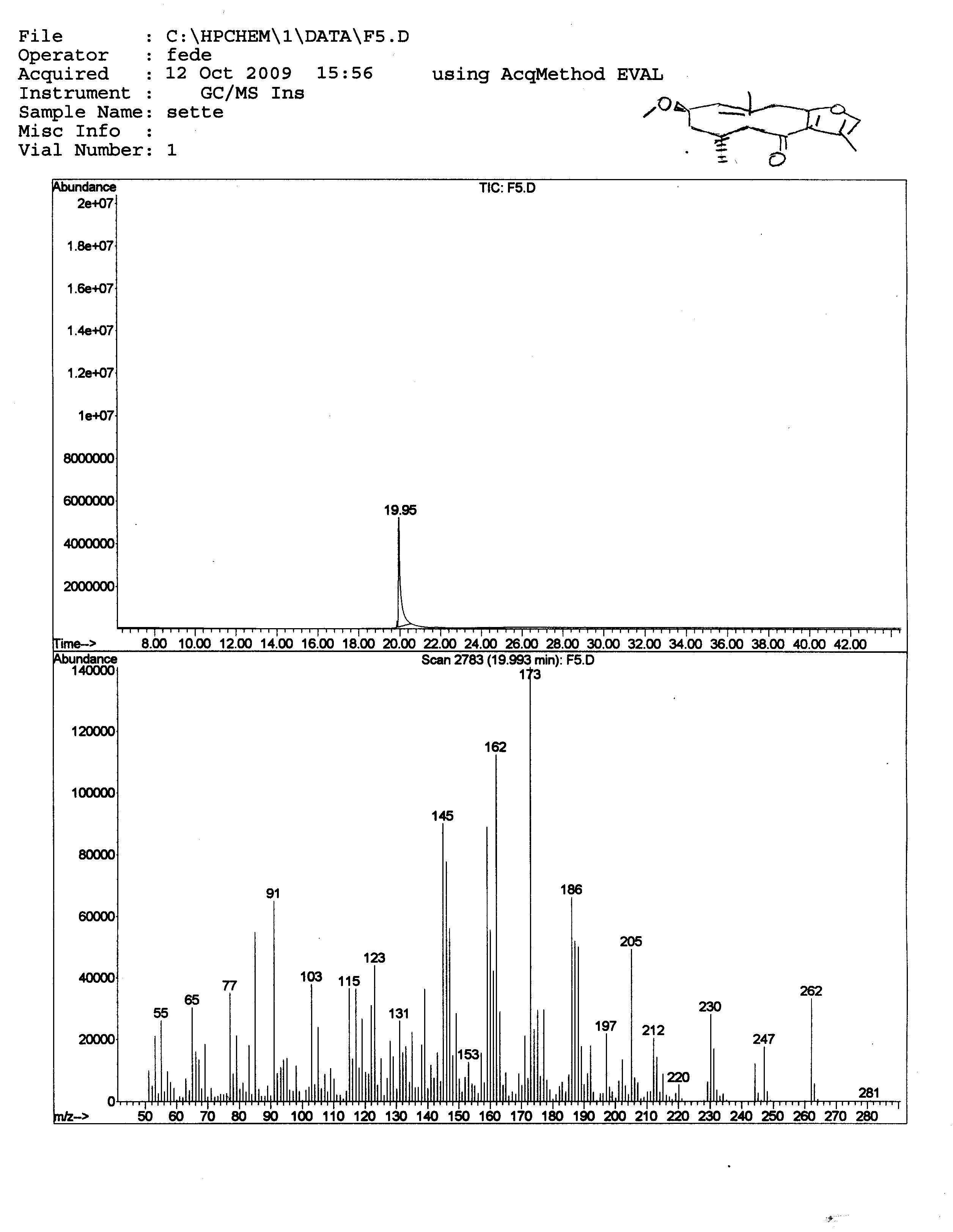
**

**Figure S2:** TLC plates of pure isolated compounds. **a)** Eluent: CH_2_Cl_2_-Acetone 0.1%. **b**) CH_2_Cl_2_-Acetone 1%. Spots were visualized after staining with p-anisaldheyde-H_2_SO_4_-EtOH (1:1:98)

Rf: **1**: 0.69 ; **2**: 0.62 ; **3**: 054; **4**: 0.42.

| 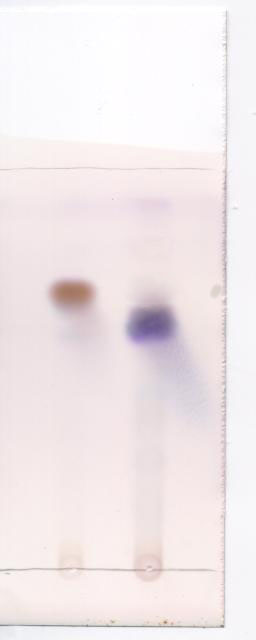  **1 2**  **a)** | 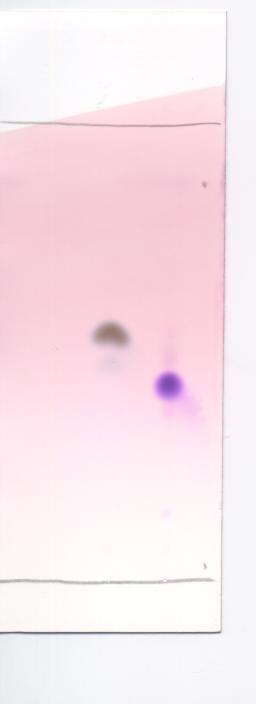  **3 4**  **b)** |
| --- | --- |

**Figure S3:** NMR spectra con isolated compounds. **a**) Compound **1**; **b)** Compound **2**; **c)** Compound **3**; **d**) Compound **4.**

1. **
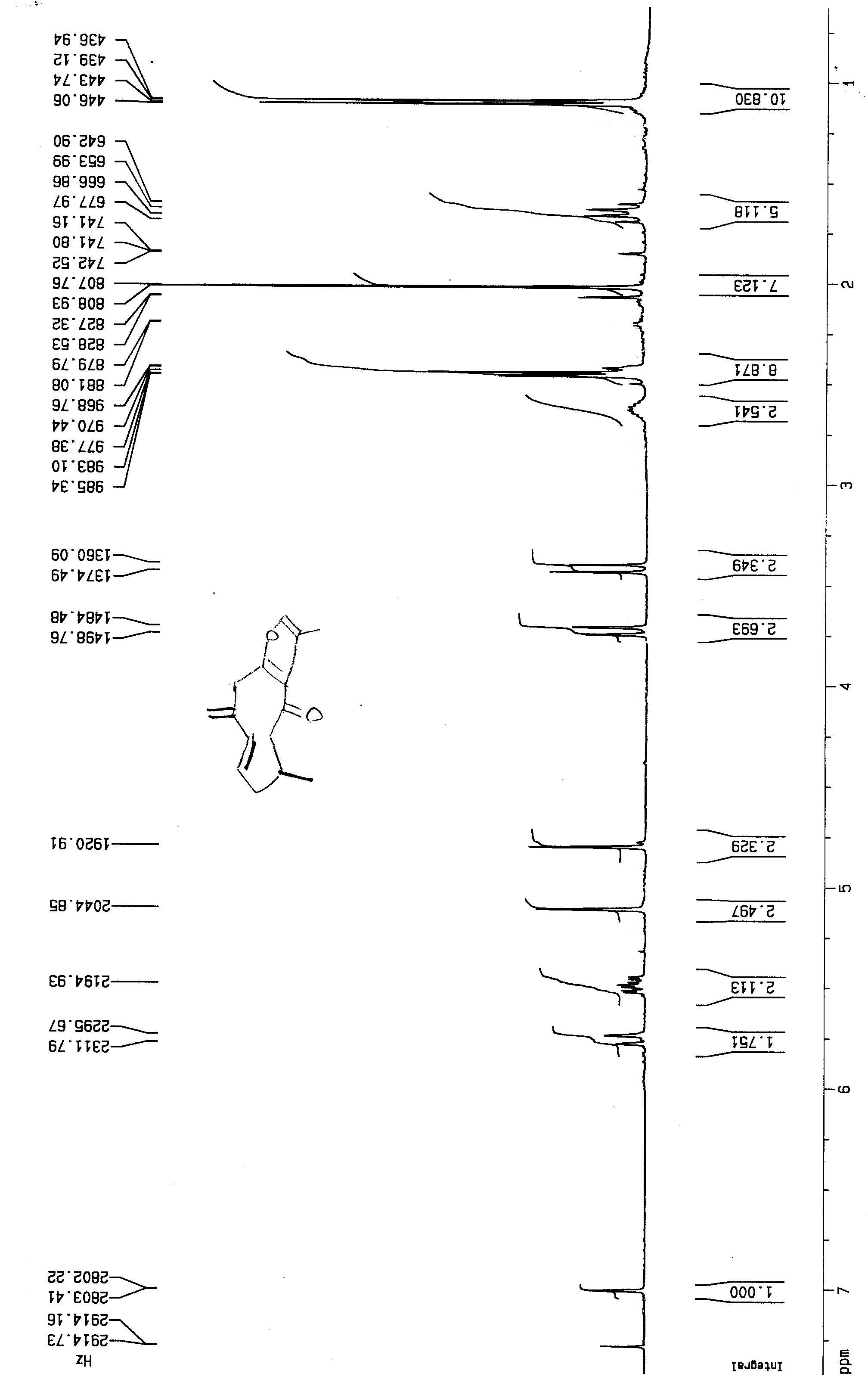
**
2. **
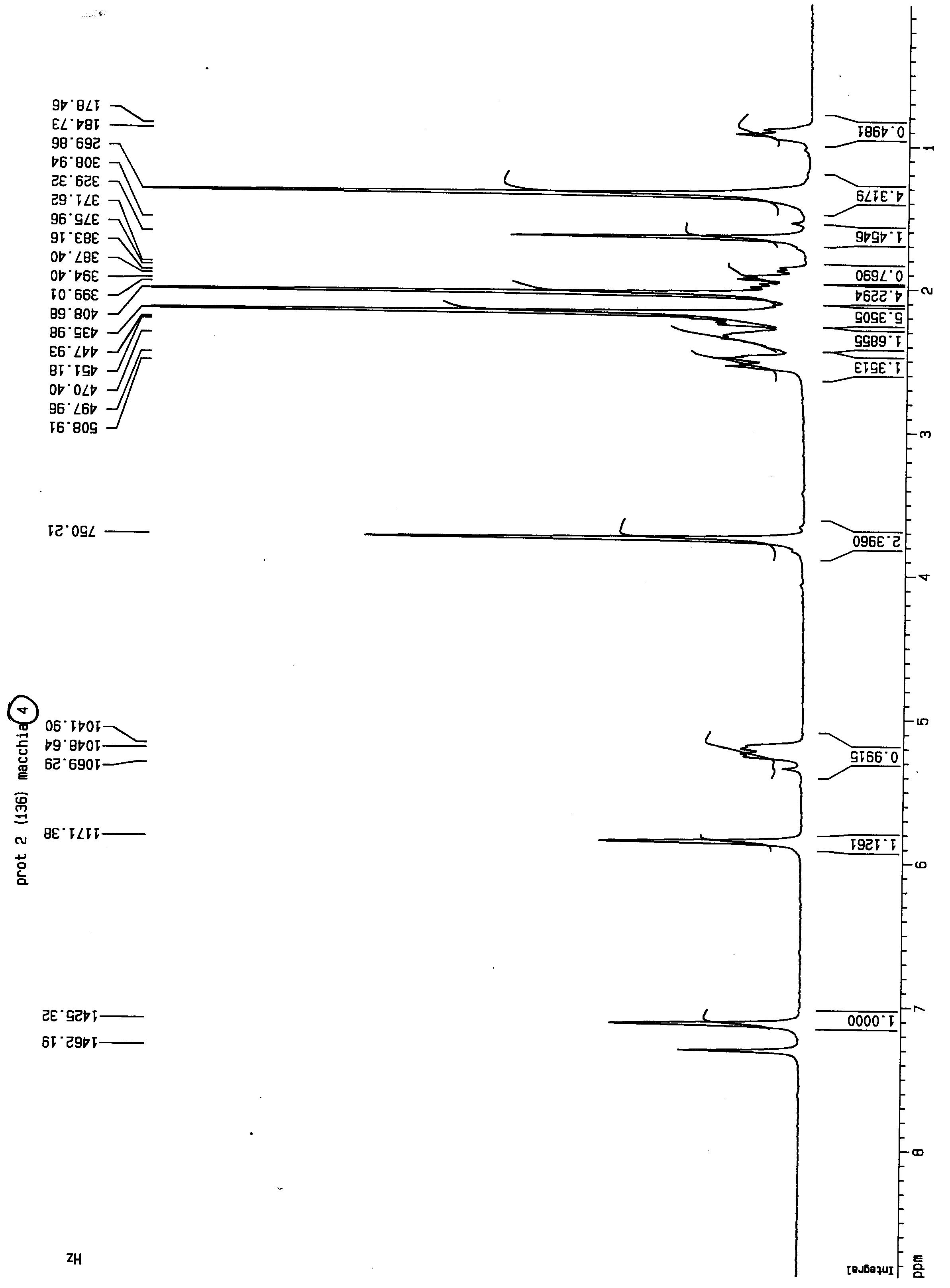
**
3. **
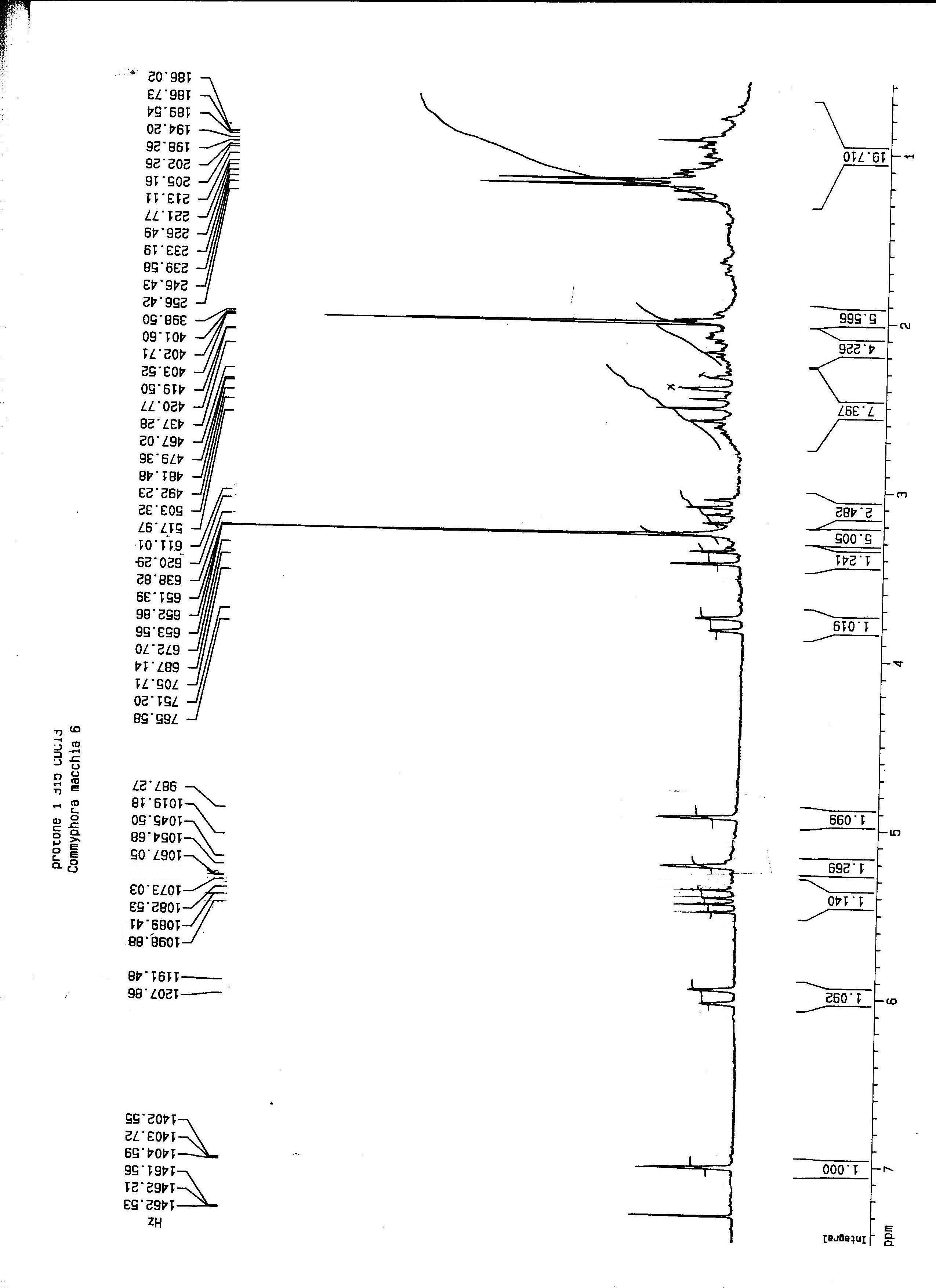
**
4. **
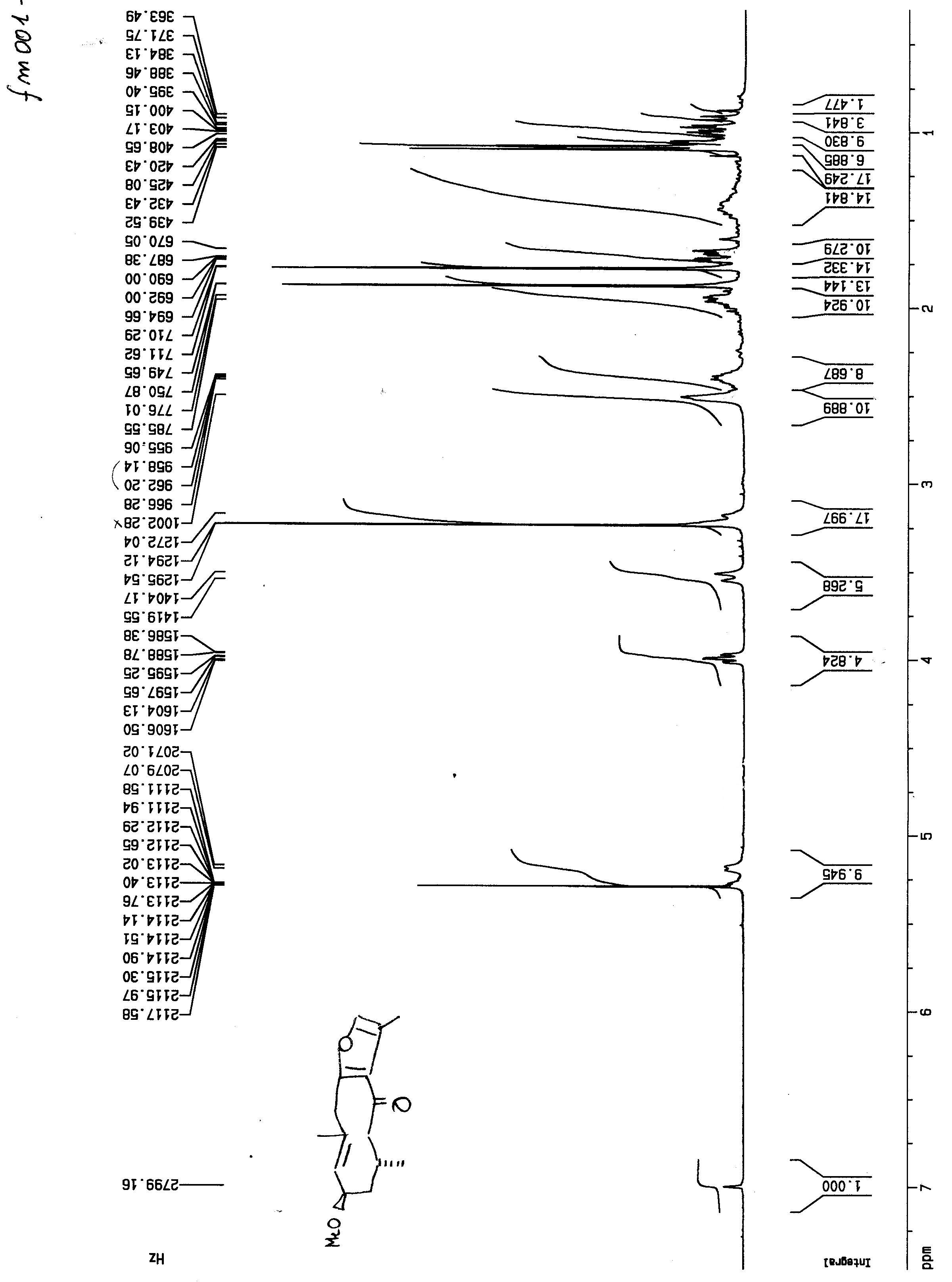
**

**Figure S4.** Verify3D plot of 5-LOX model.
